# Randomised controlled trial of an intervention to improve parental knowledge and management practices of fever

**DOI:** 10.1186/s12887-019-1808-9

**Published:** 2019-11-19

**Authors:** M. Kelly, L. Sahm, S. McCarthy, R. O’Sullivan, A. Mc Gillicuddy, F. Shiely

**Affiliations:** 1 Pharmaceutical Care Research Group, School of Pharmacy, University College, Cork, Ireland; 2 Trials Research and Methodologies Group (TRAMS), HRB Clinical Research Facility, Mercy University Hospital, Cork, Ireland; 3 Department of Pharmacy, Mercy University Hospital, Cork, Ireland; 4 Department of Pharmacy, Cork University Hospital, Cork, Ireland; 5 School of Medicine, University College Cork, Cork, Ireland; 6 National Children’s Research Centre, Dublin, 12 Ireland; 7 School of Public Health, University College Cork, Cork, Ireland

**Keywords:** Fever, Knowledge, Parent, Randomised controlled trial, Information leaflet

## Abstract

**Background:**

We know that parents require resources which can assist them to improve fever knowledge and management practices. The purpose of this study, using an RCT, was to examine the effectiveness of an information leaflet at increasing parental knowledge of fever, specifically temperature definition.

**Methods:**

A prospective, multi-centre, randomised, two-parallel arm, controlled trial with blinded outcome ascertainment was conducted. Parents presenting at purposively selected healthcare facilities who had a child aged ≤5 years of age were invited to participate. An information leaflet for use in the trial was designed based on previous studies with parents. Parents in the intervention arm read an information leaflet on fever and management of fever in children, completed a short questionnaire at Time 1 (T1) and again 2 weeks after randomisation at Time 2 (T2). Parents in the control arm did not receive the fever information leaflet but completed the same questionnaire as the intervention arm at T1 and againat T2. The primary outcome was the correct definition of fever (higher than ≥38 °C).

**Results:**

A total of 100 parents participated in the study at T1. A greater proportion of the intervention group (76%) than the control group (28%) selected the correct temperature (≥38 °C) at T1. 76% of the intervention arm correctly identified “higher than ≥38°C” as the temperature at which a fever is said to be present compared to 28% of the control arm. After 2 weeks, there was an increase of 6% of parents in the intervention arm (increase to 82.4%) who gave the correct temperature compared to just a 2.8% increase in the control arm (increase to 30.8%). Univariate logistic regression showed that parents in the intervention arm were significantly more likely to give the correct answer at both time-points (T1: OR 8.1; CI 95% 3.3–19.9: *p* < 0.01; T2: OR 10.5; CI 95% 3.4–32.0: *p* < 0.01).

**Conclusions:**

Our RCT of this simple educational intervention has been shown to improve parental understanding of fever knowledge and correct management strategies. Education interventions providing simple, clear information is a key step to decreasing parental mismanagement of fever and febrile illness in children.

**Trial registration:**

ClinicalTrials.gov NCT02903342, September 16, 2016, Retrospectively registered.

## Background

We know that parents require resources which can assist them to improve fever knowledge and management practices, and this is supported in the literature [1–6]. Fever is one of the most common childhood symptoms [3, 5, 7–11] and is defined as a temperature of greater than 38 °C [12, 13]. Fever is usually a self-limiting symptom with very low rates of associated serious illness [14–17].

Despite the benign nature of fever, parents often become alarmed and very concerned [4, 18–27]. Previous literature has demonstrated that this often leads to; aggressive treatment strategies; unnecessary and incorrect use of antipyretics [28–34]; and parents seeking medical advice [13, 19, 32, 35–38]. Consequently, fever is one of the most common reasons for consultations with healthcare professionals (HCPs) [5, 7, 39–45]. Many of these consultation could be avoided if there was clarity on fever definition and fever management practices.

The management of fever by antipyretics is acceptable, when there is associated signs of distress [8]. In medical practice alternating antipyretics is deemed necessary when fever occurs with other symptoms (e.g. otitis media, pain), however, guidelines suggest that antipyretics should not be alternated to manage fever in the absence of distress [8, 46–48]. The inherent problem with alternating antipyretics is that sometimes, a concerned parent may become confused as to what antipyretic they last administered and this may lead to dosing errors. Dosing regimens with antipyretics can lead to confusion as although similar, maximum numbers of administrations per day differ. For example, in Ireland, for a child aged 7–9 years, Calpol (Paracetamol) is administered up to 4 times per day while Neurofen (Ibuprofen) is administered up to three times per day. This can lead to possible safety issues when alternating antipyretics. HCPs, especially general practitioners (GPs) are giving appropriate information when they suggest alternating in specific cases (e.g. fever accompanied with distress despite monotherapy) [8, 49]. However, our previous research has shown that parents alternate between antipyretics both with and without the presence of distress [6]. Manufacturers have attempted to address the dangers associated with overdosing children with antipyretics by adjusting dosing schedules to include narrower age bands and including doses calculated based on weight. This serves to underpin the problems with unintentional overdose with antipyretics. However, this does not address the use of antipyretics when there is no need. The purpose of our study was thus to address this need. Our prior research has shown that parents have requested accessible, consistent and reliable information resources to be made available to them [1, 2, 6, 50], with a particular desire for paper-based information resources [1, 6]. We know that educational interventions increase parental knowledge and decrease clinic visits and consultations for fever and other minor illnesses [51–59]. thus potentially easing the burden on HCPs and providing a cost saving for the health service as well as the family seeking medical help [60].

### Aim of study

The primary aim of this study was to examine the effectiveness of an information leaflet at increasing parental knowledge of fever, specifically temperature definition. Secondary outcomes were improvements in understanding of preferred management practices for antipyretic use and tepid sponging and knowledge retention after 2 weeks.

## Methods

### Study design

A prospective, multi-centre, randomised, two-parallel arm, controlled trial with blinded outcome ascertainment.

### Study sample

Parents presenting at purposively selected GP practices, urgent and emergency care treatment centres and pharmacies in Cork, Ireland were invited to participate. The locations were selected to maximise population variation. A total of two GP practices, two urgent and emergency care surgeries and two community pharmacies participated. Any person attending these locations who had at least one child aged ≤5 years of age was invited to participate. Potential participants were approached by MK only, a research pharmacist, not involved in their care. Utilising one researcher for data collection reduces the chances of observer bias. They were asked if they had a child aged ≤5 years of age. If participants indicated that they had a child of this age, the study was explained in detail to them, they were given an information leaflet about the study and written informed consent was obtained. Each participant was given a study number, which were consecutively assigned. Following this process, MK opened the envelope corresponding to the participant’s study number and parents were randomised to either the control or intervention arm of the study. All participants who consented to this study were parents and will be referred to as parents throughout.

### Information leaflet design

An information leaflet (Additional file 1: Appendix 1) for use in the trial was designed based upon our two previous studies with parents [1, 6]. Previous research included conducting a large scale quantitative study with parents using a questionnaire to gather data [6]. The questionnaire was developed and used in previous research [13]. We modified the questionnaire to reflect custom and practice in Ireland and piloted with a sample of five parents. It consisted of 38 questions with sub-themes (Additional file 1: Appendix 2). The questionnaire assessed parental knowledge, help-seeking behaviours and expectations, needs for additional resources, fever management practices, use of pharmaceutical products, and concerns, attitudes and beliefs.

To develop the leaflet we sought input from five stakeholders including parents, pharmacists, doctors and nurses was also sought before the final leaflet was designed. The National Adult Literacy Agency (NALA) reviewed and revised the leaflet to ensure the language used was presented in a non-technical manner and was easily understood [61]. The leaflet meets plain English standards and carries the Plain English Mark [61]. Parents did not receive any reward for participation.

### Intervention arm

Parents in the intervention arm of the study were given the information leaflet. After reading the leaflet, they then completed an initial questionnaire The questionnaire consisted of twenty questions – demographics, questions on knowledge of fever and antipyretic use, and measures of satisfaction with the fever information leaflet (See Additional file 1: Appendix 3). Parents were asked to provide contact details and a preferred contact time to participate in a similar follow up questionnaire (Additional file 1: Appendix 4) 2 weeks after randomisation. Parents were contacted by telephone 2 weeks later, at which time the follow-up questionnaire was completed. Parents were not given a specific date or time to ensure true learning effects from the leaflet were observed. At randomisation, if parents indicated that they would prefer to do the questionnaire at home and return by post, they were provided with stamped addressed envelopes to do so. All parents were contacted a maximum number of three times. If after three attempts to contact them, no contact was made, then they were recorded as a non-responder to part two of the study.

### Control arm

Parents in the control arm were asked to complete a short questionnaire. The questionnaire was the same as the intervention arm in all respects but excluded the questions on the fever information leaflet (Additional file 1: Appendix 3). Parents were asked to provide contact details and a preferred contact time to participate in a similar follow up questionnaire (Additional file 1: Appendix 4) 2 weeks after randomisation. Parents were contacted by telephone approximately 2 weeks later, at which time the questionnaire was completed. Parents were not given a specific date or time to ensure true learning effects from the leaflet were observed.

### Study outcomes

The primary outcome was the correct definition of fever (higher than ≥38 °C as the temperature at which a fever is said to be present). Secondary outcomes were improvements in understanding of preferred management practices for antipyretic use and tepid sponging. The tertiary outcome was knowledge retention after 2 weeks.

### Sample size

A sample size calculation was conducted based on results from a previous study examining parental fever knowledge [6]. We previously showed that 37% of parents were able to correctly identify the correct temperature at which their child could be said to have a fever. We expected this to increase to 64% after the intervention. Therefore, with a minimum clinically important difference of 27, 80% power and a type one error rate, alpha = 0.05, we require a sample size of 100.

### Randomisation

AMG, a research pharmacist unconnected with the study, conducted the randomisation based on study number using a dedicated website [62]. Participants were allocated to one of two arms (intervention or control). The allocation sequence was concealed through the use of individual sequentially numbered opaque sealed envelopes prepared by AMG. The number on the envelope corresponded to participants’ study number. The primary researcher MK, who was involved in recruiting participants, was not involved in this step of the process. Once participants had consented to participate in the trial, they were randomly assigned to the control or intervention arm by opening the opaque sealed envelope corresponding to their study number. Neither participants nor MK were blinded to the intervention.

### Data treatment

Data were entered into a Microsoft Excel database by AMG. AMG blinded participant allocation from MK until analysis was completed. Data were analysed by MK on an intention-to-treat basis, using SPSS version 22.0 (SPSS, Inc., Chicago IL). Categorical variables were described by the count and proportion in each category. Continuous variables were described by means and standard deviations (SDs), or by medians and inter-quartile ranges (IQRs), depending on whether they were normally distributed or not. Bivariate associations between categorical variables were assessed using Pearson’s Chi-square test. *P*-values < 0.05 were considered to be statistically significant, given a null hypothesis of independence. Univariate logistic regression was conducted to estimate associations, reported as odds ratios (ORs) and 95% confidence intervals (CIs), between the control and intervention arms. The dependant variable was the primary outcome, correct definition of fever. Multiple logistic regression was not deemed necessary as previous research showed no associations with key sociodemographic factors [6].

### Reporting

Reporting is in accordance with the Consolidated Standards of Reporting Trials (CONSORT) checklist [63].

### Trial registration

Trial registration: ClinicalTrials.gov NCT02903342, 16 September 2016, Retrospectively registered.

## Results

### Participation and demographics

**Fig. 1:**
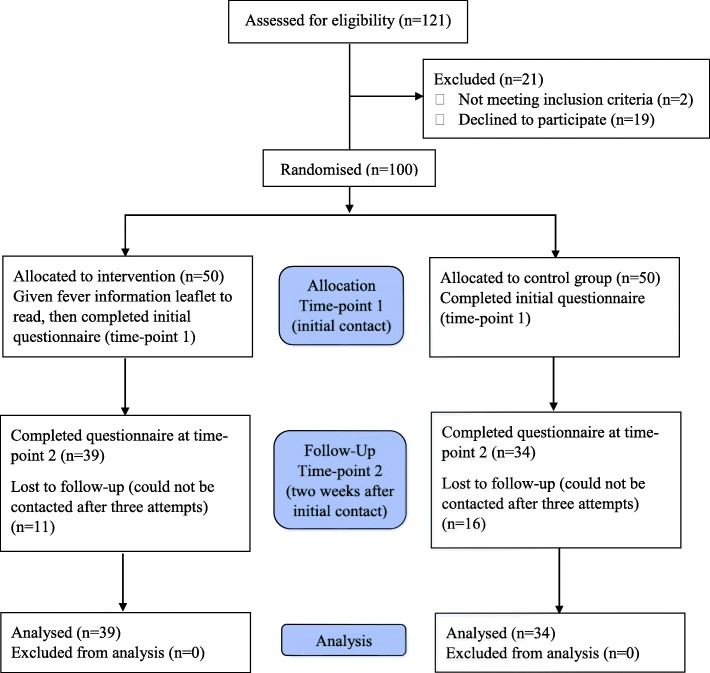
Flow chart of participant enrolment, allocation, follow up and analysis

Data were collected during July and August 2016. One hundred and twenty one parents were approached to take part in the study and 100 parents consented to participate in phase 1 of the study with 50 parents in each arm.. For the follow-up, at 2 weeks post intervention, 39 of the control arm and 34 parents from the intervention arm had been successfully contacted, a 73% follow-up response rate. A flow diagram describing participation is shown in Fig. 1.

The demographics of the included parents at T1 and T2 are shown in Table 1 below. For the most part, the intervention and control arms were similar. However, there are some differences between the two groups at T1 and T2 in education levels and marital status. The proportion of Irish people in the intervention and control groups also differed slightly. At T1 only, there was a higher proportion of females in the control group compared to the intervention group. Parents were randomised using sequentially numbered opaque sealed envelopes so given that the groups were randomised, it does not affect the results or conclusions of our study.

**Table 1:** Parent demographic information at T1 and T2

		Time Point 1		Time Point 2	
		Intervention Arm (*n* = 50)	Control Arm (*n* = 50)	Intervention Arm (*n* = 34)	Control Arm (*n* = 39)
Age of participant (yrs)	Range	24–48	22–43	24–48	24–43
	Mean/median	35.5	34	35.5	34
	IQR	33.0–38.0	31.8–37.0	33.0–38.3	33.0–37.0
Gender (%)	Female	88	94	91.2	92.3
	Male	12	6	08.8	7.7
Education (%)	Tertiary	72	81.6	67.6	81.6
	Secondary	24	16.3	26.5	15.8
	Primary	4	2	5.9	2.6
Nationality (%)	Irish	80	92	85.3	92.3
	British	4	2	2.9	2.6
	Polish	2	2		2.6
	American	4		2.9	
	French	2		2.9	
	Mauritian	2		2.9	
	Ecuadorian	2			
	Lithuanian	2			
	Australian	2		2.9	
	Bangladesh		2		2.6
	Italian		2		
Marital Status (%)	Married	78	74	79.4	76.9
	Cohabiting	2	20	11.8	20.5
	Single	8	6	5.9	2.6
	Civil P’ship	12		2.9	
Number of children	Range	1–5	1–6	1–4	1–6
	Mean	2.06	2.1	2.0	2.1
Age of children (yrs)	Range	2 wk-29 yrs	7wk-16	2wk-29 yrs	8wks-
	Median	3	yrs	2.5	16 yrs
	IQR	1–6	3 1–5.62	10.4mth-4.5 yrs	3 1–6

A number of different healthcare and/or insurance types were selected by parents, often with different children in the same family having different types of healthcare provision. Types of healthcare provision included: medical cards (full publically funded health care and medicines– income dependant), GP visit cards (publically funded cost of GP care only – income dependant), GP visit card for children aged ≤5 years of age (publically funded GP care for children under 5 years regardless of income) and private patients (private health insurance). Similarly, a variety of care choices were selected by parents, often with different children in the same family receiving care from one or more providers including; parent, family member, crèche, childcare centre, school, nanny, au pair.

#### Primary outcome analysis

Results show that 76% of the intervention arm correctly identified “higher than ≥38°C” as the temperature at which a fever is said to be present compared to 28% of the control arm. After 2 weeks, there was an increase of 6% of parents in the intervention arm (increase to 82.4%) who gave the correct temperature compared to just a 2.8% increase in the control arm (increase to 30.8%).

Univariate logistic regression showed that parents in the intervention arm were significantly more likely to give the correct answer at both time-points (T1: OR 8.1; CI 95% 3.3–19.9: *p* < 0.01; T2: OR 10.5; CI 95% 3.4–32.0: p < 0.01). There was no association between the primary outcome, correct definition of fever, and any of the demographic factors.

### Secondary outcome analysis

#### Use of medication

Out of 100 parents, 56% in the intervention arm would use medication at temperatures ≥38 °C regardless of the child’s distress, compared to 84% of parents in the control arm (Table 2). 38.0% in the intervention arm would use medication at ≥38 °C when the child is distressed compared to 14% in the control group. Six percent of intervention arm parents would not give medication at the correct temperature at which it is advised, compared to 2% in the control arm. After 2 weeks, while little has changed in the control arm, there is a notable change in the intervention arm, with a clear increase from 38 to 47% of the number of parents waiting for signs of distress in the child before administering medication. Using logistic regression, the intervention arm were significantly more likely to only use medication at this temperature if the child was also distressed at both time-points (T1: OR 3.8, CI 95% 1.1–10.0, *p* = 0.008; T2: OR 4.1, CI 95% 1.4–11.7 *p* = 0.009).

**Table 2:** Medication use at temperatures ≥38 °C in the intervention and control arms at the two time points

	T1 (*n* = 100)		T2 (*n* = 73)	
	Intervention Arm (%)	Control Arm (%)	Intervention Arm (%)	Control Arm (%)
Use of medications regardless of distress	56	84	47	82
Use of medication when child is distressed	38	14	47	18
No medication	6	2	6	0

#### Alternating medication

The most obvious change from time on to time two is that no parent was unsure (Table 3). Of note is the substantial differences between the intervention and control arms in the proportion of parents using alternating pyretics, regardless of distress. After 2 weeks, there is a notable increase, 44 to 56%, in the proportion of parents in the intervention arm who would alternate medication when the child is distress.

**Table 3:** Parents’ opinions on alternative antipyretics at temperature ≥ 38 °C in both arms of the study at time point 1 and 2

	T1 (*n* = 100)		T2 (*n* = 73)	
	Intervention Arm (%)	Control Arm (%)	Intervention Arm (%)	Control Arm (%)
Alternating antipyretics regardless of distress	26	54	15	51
Alternating medication when child is distressed	44	16	56	21
No medication	22	24	29	28
Unsure	8	6	0	0

Logistic regression analysis showed that the intervention arm were more likely to select that alternating antipyretics was effective if the child had a temperature and was distressed at both time-points (T1: OR 4.1, CI 95% 1.6–10.6, *p* = 0.003; T2: OR 4.9, CI 95% 1.8–13.8, *p* = 0.002).

#### Using medication together

In the intervention arm, only 10.0% thought that using antipyretics together was the most effective way to manage fever, 60.0% did not think it was the most effective way to manage fever, while 30.0% were unsure. In the control arm, 22.4% of parents thought that using antipyretics together was the best way to manage fever, 63.3% of parents did not think it was the most effective way to manage fever, while 14.3% were unsure.

#### Use of tepid sponging

There are large proportionate differences between knowledge on tepid sponging between both the intervention arm and the control arm at both time points (Table 4). There is a notable change after 2 weeks from 24% unsure to 0% unsure in the intervention arm. A similar change occurred for the control arm.

**Table 4:** Parents’ beliefs on the effectiveness of tepid sponging for treating fever

Tepid Sponging is Effective	T1 (*n* = 100)		T2 (*n* = 73)	
	Intervention Arm (%)	Control Arm (%)	Intervention Arm (%)	Control Arm (%)
Agree	4	52	3	62
Disagree	72	18	97	36
Unsure	24	30	0	3

Following logistic regression, the intervention arm were significantly more likely to think that tepid sponging was not an effective way to manage fever at time-point 1 (OR 11.7, 95% CI 4.5–30.3, *p* < 0.01). This was similar at time-point 2 (OR 58.9, 95% CI 7.2–487.5 *p* < 0.01).

#### Satisfaction with the leaflet

The majority of parents (88.0% *n* = 44) found the information leaflet useful. Some comments about the leaflet included *“very informative for parents”* (questionnaire 74); *“When your child is in the middle of a fever-leaflet would be great to consult”* (questionnaire 11). Almost half of parents (46.0%) thought the leaflet was easy to remember, while 58.0% stated it had information that was helpful and 58.0% thought the leaflet was easy to read.

Parents also suggested further information for inclusion, e.g., when to consult at an emergency department, what to do if a child has a febrile seizure, a stepwise approach describing what to do when a child has a fever.

## Discussion

The findings from our RCT of an educational intervention showed that an information leaflet for parents increases correct definition of fever temperature and decreases incorrect management practices. The increase in knowledge was sustained, and further improved, up to 2 weeks after administration of the leaflet. Parents who received a copy of the leaflet before completion of the questionnaire were more likely to select the correct temperature definition for fever, less likely to believe that using antipyretic medication together is more effective, and more likely to think that alternating antipyretics is effective only if the child is still distressed. These parents were also less likely to use medication unless their child had a temperature and was simultaneously distressed, and less likely to use tepid sponging. We found that satisfaction with the leaflet was high.

Parents have repeatedly highlighted a need for information resources [1, 2]. The current research suggests that this information leaflet can empower parents so that they can take an informed approach to the care of their children when they have a fever or febrile illness, similar to previous research [64]. We know that increasing parents’ health knowledge and health literacy is important as health literacy and good health outcomes are linked [65]. Previous research has suggested that limited health literacy can lead to higher rates of hospitalisation, poorer self-management skills and lower use of preventative services [66–71]. What is less clear is how that information should be imparted. What we have shown is that a simple information leaflet is an effective means of addressing this need in the area of treating fever and febrile illness in children. It is reported that retention of information following consultations can be low [72], sometimes as low as 20%, however with the incorporation of visual or written information, this can increase to 50% [50]. Our study, though not undertaken at a consultation, has shown that retention of information from a simple information leaflet is extremely high. In fact at 2 weeks post receiving the information leaflet, there was an increase in knowledge level. Further studies are necessary to establish the effect of this information leaflet during a GP consultation. We know that previous research has demonstrated that information resources could potentially decrease consultations with HCPs [52, 57, 74–77]. Any possible reduction in presentation rates could decrease pressure on healthcare services which we know are at unsustainable levels in Ireland, on a background of an increasing older population and a GP rate of 2.7 per 1000 population [78–81]. Furthermore, the accessible nature and location of pharmacies could provide a suitable point of contact and distribution area for these leaflets. Further research to establish the leaflet’s viability is needed in this setting.

## Strengths and limitations

The strength of this study is that it was a randomised controlled trial, the gold standard methodology for establishing causation. Therefore any findings we infer are deemed to be strong. The leaflet utilised was approved by NALA, thereby reaching as many parents as possible. There were a number of limitations with this study. The leaflet was not individualised. While a strength of the leaflet was that it was generated based on results from our previous studies [1, 2], it did not take into consideration what was already known by parents nor did it take their specific needs into consideration [82]. This may serve as both an advantage and a disadvantage. From an advantageous point of view, the included information is what parents in general want included and therefore is suitable for all parents, a disadvantage is that it is not personalised (e.g. availability in other languages). Furthermore, this trial used a closed question questionnaire design therefore answers may not reflect the answers which parents may freely give, should they have had free choice [52]. There is also a risk that parents in the intervention arm at time-point 1 transferred information from the leaflet to the questionnaire without fully understanding or changing their understanding of the topic. Similarly, social desirability risk cannot be eliminated as parents were aware of the profession of the researcher. There was a drop out between time period one and two, 11 people from the intervention arm and 14 people from the control arm, therefore our sample size calculation does not hold for time period two. It is therefore necessary to interpret this phase of the results with caution. Finally, despite sampling in six purposively selected locations over several days, the included sample are mainly female with high education levels. However, previous research has suggested that mothers often provide the majority of care for children so we do not feel that this has biased our results in this study [45]. The included sample are from the general Cork area with a large proportion of Irish parents, therefore, it could be argued that results indicated in this study are not generalisable to other cultures.

## Conclusions

Our prior research has shown that knowledge of the correct definition of fever is key to parents’ management practices for fever. This information leaflet increased the number of parents’ correctly answering questions about managing fever and also showed that information retention is high after 2 weeks. Educational interventions providing simple, clear information is a key step to decreasing concern and anxiety and increasing parents’ confidence in treating fever and febrile illness in their children [73].

## Supplementary information

**Additional file 1:** **Appendix 1.** Information Leaflet (Managing Fever in Children: Advice for Parents and Carers). **Appendix 2.** Pre-Intervention Questionnaire to Develop the Information Leaflet (Parents’ Knowledge, Attitudes and Practice in Childhood Fever). **Appendix 3.** Intervention and Control Questionnaires T1. **Appendix 4**. Intervention and Control Questionnaires T2

**Additional file 2.** CONSORT Checklist

## Data Availability

For questionnaire use or reproduction, please contact the original authors as per reference number 90. The datasets used and/or analysed during the current study are available from the corresponding author on reasonable request.

## Abbreviations

CI: Confidence Interval
CONSORT: Consolidated Standards of Reporting Trials
GP: General Practitioner
HCP: Health Care Professional
NALA: National Adult Literacy Agency
OR: Odds Ratio
RCT: Randomised Controlled Trial
SD: Standard Deviation
T1: Time 1
T2: Time 2
