# Supplementary material for: Randomised controlled trial of an intervention to improve parental knowledge and management practices of fever
Source: BMC Pediatr. 2019 Nov 19;19:447. doi: 10.1186/s12887-019-1808-9 (PMC6863059; doi:10.1186/s12887-019-1808-9)
Supplement: Supplementary file 1 — Additional file 1: Appendix 1. Information Leaflet (Managing Fever in Children: Advice for Parents and Carers). Appendix 2. Pre-Intervention Questionnaire to Develop the Information Leaflet (Parents’ Knowledge, Attitudes and Practice in Childhood Fever). Appendix 3. Intervention and Control Questionnaires T1. Appendix 4. Intervention and Control Questionnaires T2 [file 12887_2019_1808_MOESM1_ESM.docx]

**Appendices**

**Appendix 1**

**Information leaflet**

Appendix 2

**Appendix 3**

**Intervention group**

**Control group**

Appendix 4

**Intervention group**

**Control group**
